# Strain-tunable electronic and optical properties of BC_3_ monolayer

**DOI:** 10.1039/c7ra10570c

**Published:** 2018-01-05

**Authors:** Yang Zhang, Zhi-Feng Wu, Peng-Fei Gao, Dang-Qi Fang, Er-Hu Zhang, Sheng-Li Zhang

**Affiliations:** Department of Applied Physics, School of Science, Xi'an Jiaotong University Xi'an 710049 China yzhang520@mail.xjtu.edu.cn zhangsl@mail.xjtu.edu.cn

## Abstract

Two-dimensional layered nanostructures with unique electronic and optical properties may hold great potential in nanoelectronics and optoelectronics applications. In this work, structural stability, elastic, electronic, and optical properties of BC_3_ monolayers have been investigated using a first-principles study. The BC_3_ monolayer can be regarded as a series of hexagonal C rings with the connections of B atoms, which has been tested to be highly dynamically stable. The in-plane stiffness is 316.2 N cm^−1^, potentially rivalling graphene. A screened hybrid density functional HSE06 is used to calculate the electronic and optical properties. It is found that the BC_3_ monolayer is an indirect band gap semiconductor with a moderate gap energy of 1.839 eV. Spatial charge distribution to the valence band maximum and the conduction band minimum is analyzed to explore the origin of indirect band gap features. By calculating the complex dielectric function, optical properties considered as excitonic effects are discussed. Besides, the effects of various in-plane strains on electronic and optical properties are explored. Our results of good structural stability, moderate and tunable band gap, and strain-controllable optical properties suggest that the BC_3_ monolayer holds great promise in the applications of nanoelectronic and optoelectronic devices.

## Abstract 2

The BC_3_ monolayer holds great promise in the applications of nanoelectronic and optoelectronic devices due to its good structural stability, moderate and tunable band gap, and strain-controllable optical properties.

## Graphical abstract

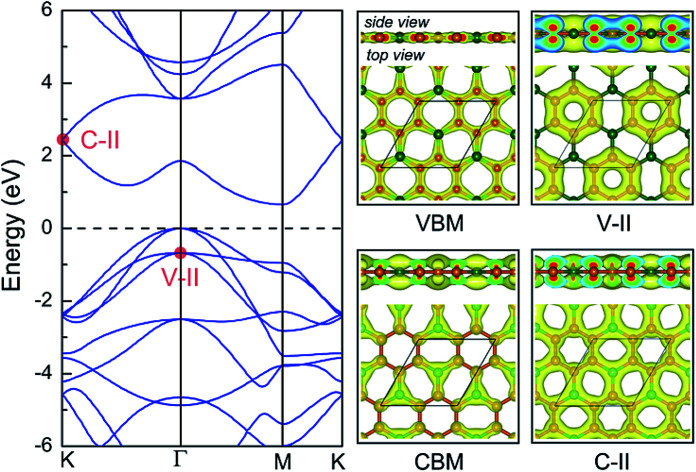

## Introduction

Two-dimensional (2D) atomic-layer nanostructures, such as graphene,^1,2^ silicene,^3^ borophene,^4,5^ and phosphorene,^6,7^ have attracted extensive research efforts in recent years for their potential applications in future electronics. For high-performance electronic devices such as field-effect transistors, a moderate electronic band gap and a reasonably high carrier mobility of the channel material are indispensable.^8,9^ Graphene and silicene have a very high Fermi velocity,^10,11^ however, their intrinsic band dispersions are gapless, which limits the direct utilization in nanoelectronic and nanophotonic devices. Phosphorene, a new atomically thin single-layer with a moderate band gap, good charge carrier mobility, and high on/off ratio, holds great promise to replace silicon in nanoelectronics. But, phosphorene has a fatal disadvantage in its structural stability. It can react with water vapor and oxygen assisted by visible light to degrade within hours.^12,13^ Therefore, finding new 2D layered nanostructures with moderate band gaps and good structural stability is critical, and can extend the possible electronic applications.

Within the hexagonal honeycomb structure, BC_3_ monolayer is another typical layered-structure with excellent crystalline quality and has been synthesized experimentally on a NbB_2_ (0001) surface by a carbon-substitution technique in a boron honeycomb.^14–16^ Subsequently, theoretical calculations predict that the BC_3_ monolayer can be stable on the NbB_2_ (0001) surface by analyzing the thermodynamic stability and chemical bonding.^17^ First-principles studies within a local density approximation or generalized gradient approximation show that the BC_3_ monolayer is an indirect band gap semiconductor with the gap energy of about 0.5–0.76 eV.^18–23^ By using surface modifications, and introducing vacancy and antisite defects, the electronic properties of BC_3_ monolayer can be tuned significantly. For examples, surface hydrogenation can induce semiconductor to metal transitions.^20,24^ Mono- and di-vacancies can result in the BC_3_ monolayer exhibiting magnetic properties.^21^ Moreover, by cutting or rolling the BC_3_ monolayer into nanoribbons or nanotubes, the electronic properties vary significantly, depending on the structural types.^19,25,26^ It has been found that the BC_3_ nanotubes are semiconductors with gaps of about 0.4–0.9 eV, whereas the BC_3_ nanoribbons are semiconductors or metals depending on the edge atoms. However, these results above are based on the calculations of density functional theory which has a disadvantage of underestimating the band gap of semiconductor.

For the practical applications in electronic devices, tailoring electronic properties is highly desirable. One of richer possibilities of the band structure engineering should be generated through applying in-plane strains to 2D nanostructures.^27–29^ However, for the BC_3_ monolayer, strain-tunable electronic and optical properties are still unknown. In this study, we will employ the screened hybrid density functional HSE06 to systematically investigate structural stability, elastic, electronic and optical properties of the BC_3_ monolayer. Besides, the effects of various in-plane strains on the band gap and optical absorption are explored to exploiting the potential applications in nanoelectronics and optoelectronics.

## Computational methods

Our studies are performed by using first-principles study based on the spin-polarized density functional theory (DFT) within the projector augmented wave method,^30,31^ as implemented in Vienna *ab initio* simulation package (VASP).^32,33^ The generalized gradient approximation (GGA) with the functional of Perdew–Burke–Ernzerhof (PBE) is employed to describe the electron exchange–correlation interactions.^34,35^ The cut-off of plane-wave kinetic energy and the convergence of total energy are set to be 450 eV and 10^−5^ eV, which are tested to sufficiently achieve a high accuracy. All studied BC_3_ layers are modelled in a hexagonal cell and located in the *x*–*y* plane. Because of the application of periodic boundary conditions, a vacuum region over than 10 Å is applied along the *z*-axis in order to eliminate the interactions between neighbour layers. Thence, the brillouin zone integrations are approximated by 7 × 7 × 1 *k*-point meshes with gamma centered grid. Because a larger rectangular supercell size is adopted to explore the affects of in-plane strain, smaller *k*-point meshes of 7 × 5 × 1 are used. Structural relaxations are performed by computing the Hellmann–Feynman forces using conjugate gradient algorithm within a force convergence of 0.01 eV Å^−1^.^36^ In order to evaluate the structural stability of the BC_3_ monolayer, the phonon spectrum is calculated by using the PHONON software.^37^ This method has been confirmed to be valid for exploring the two-dimensional nanomaterials.^38,39^ For the investigations of electronic band structures, the screened hybrid density functional HSE06 is employed,^40^ which is demonstrated to be an accurate measure of band gaps for semiconductors.

## Results and discussion

### Structural stability and elastic property

**Fig. 1:**
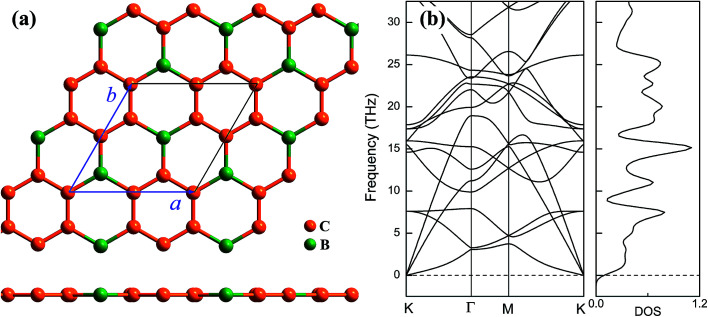
(a) Top and side views of geometrical structure and (b) phonon spectrum together with density of state (DOS) of BC_3_ monolayer.

**Fig. 2:**
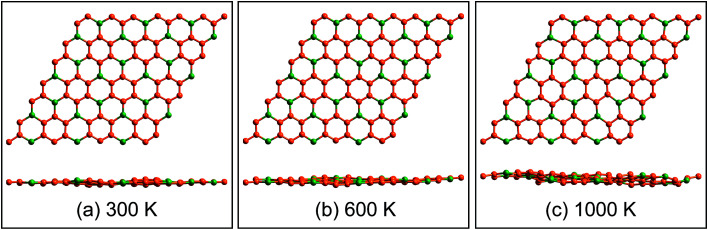
Top and side views of snapshots for the dynamic equilibrium structures of BC_3_ monolayer at the temperatures of 300 K, 600 K, and 1000 K after 10 ps *ab initio* molecular dynamics simulations.

Geometrical structure of BC_3_ monolayer is shown in Fig. 1a, which includes two B and six C atoms in a hexagonal unit cell within a space group of *P*6̄*mmm*. On the other words, the BC_3_ monolayer can be regarded as a series of hexagonal C rings with the connections of B atoms. After the fully structural optimization, all atoms prefer to sustain in a planar sheet, similar to graphene. The optimized structural parameters are list in Table 1. Due to the chemical interactions between B and C atoms, the C–C bond length of BC_3_ monolayer is slightly smaller than that in graphene.^41^ However, its lattice constant is over two times larger than that of graphene. Besides, the structural stability of the BC_3_ monolayer is explored by calculating the phonon spectrum. The corresponding results are displayed in Fig. 1b. It can be seen that there are no any imaginary modes of lattice vibrations in the whole Brillouin zone. For further examining the dynamic stability, *ab initio* molecular dynamics (MD) simulations are performed. A large 3 × 3 supercell is employed by heating the structure to 300, 600, and 1000 K within a canonical ensemble. In each case, the simulation is last for 10 ps with a time step of 1.5 fs. At the end of each simulation, the final structure is carefully examined. The snapshots viewed from different directions are presented in Fig. 2. At a low temperature of 300 or 600 K, BC_3_ monolayer could maintain its structure very well. Even at a high temperature of 1000 K, it can still withstand with slight distortions which are not sufficient to destroy the B–C and C–C bonds. Therefore, the original structure could be remained. These results of the phonon calculations and MD simulations indicate that the BC_3_ monolayer is highly dynamical stability.

**Table 1:** Properties of BC_3_ monolayer and others 2D nanostructures: lattice constant *a* (Å), bond length *l* (Å), in-plane stiffness *C* (N m^−1^), and band gap *E*_g_ (eV) using PBE functional and HSE06 method

structures	*a* (*a* = *b*)	*l*_B–C_	*l*_C–C_	*C*	*E*_g-PBE_	*E*_g-HSE06_
BC_3_	5.174	1.565	1.422	316.2	0.658	1.839
Graphene	2.467 (ref. 41)	—	1.424 (ref. 41)	340 ± 50 (ref. 42)	—	—
Graphdiyne^43^	6.89	—	—	166	0.46	0.96
BN^44^	2.51	—	—	267	—	4.61 (GW_0_)

For a two dimensional (2D) nanostructure, especially for a monolayer, the in-plane stiffness is one of the important aspects to evaluate the elastic properties.^42–45^ Although the BC_3_ monolayer has the same hexagonal symmetry and honeycomb structure with graphene, the existence of B atoms could be change the elastic properties. In order to explore that, an in-plane biaxial strain is applied to the BC_3_ monolayer. By fitting the strain energy in the range of −0.6% to 0.6%, the in-plane stiffness can be obtained according to the equation *C* = (1/*S*_0_)(∂^2^*E*/∂*ε*^2^),^43,44^ where *S*_0_ is the equilibrium area. It is found that the in-plane stiffness of the BC_3_ monolayer is 316.2 N m^−1^ (see Table 1), which is comparable to 340 ± 50 N m^−1^ of graphene,^42^ but much larger than that in others 2D monolayers, such as graphdiyne,^43^ BN,^44^ and phosphorene.^45^ Thereby, the BC_3_ monolayer could be expected to be a hard nanostructure and potentially rivals graphene. Besides, if a uniaxial strain is applied to the layer, a uniaxial in-plane stiffness can be obtained. It is found that the stiffness along the zigzag or armchair direction is 129.3 or 129.8 N m^−1^. Because of the strain energy release in the vertical direction, these stiffnesses are much smaller than the biaxial one but still larger than those of phosphorene and its analogs.^45^

### Electronic property

**Fig. 3:**
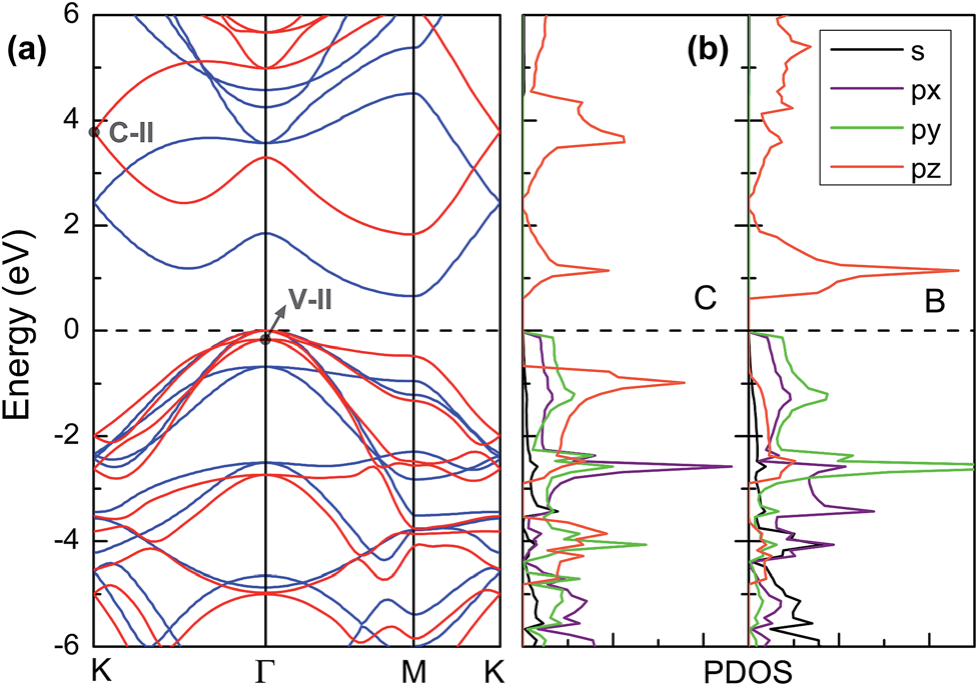
(a) Band structures calculated by using HSE06 method (red line) and PBE functional (blue line) and (b) partial density of state (PDOS). The highest occupied level is set to zero.

**Fig. 4:**
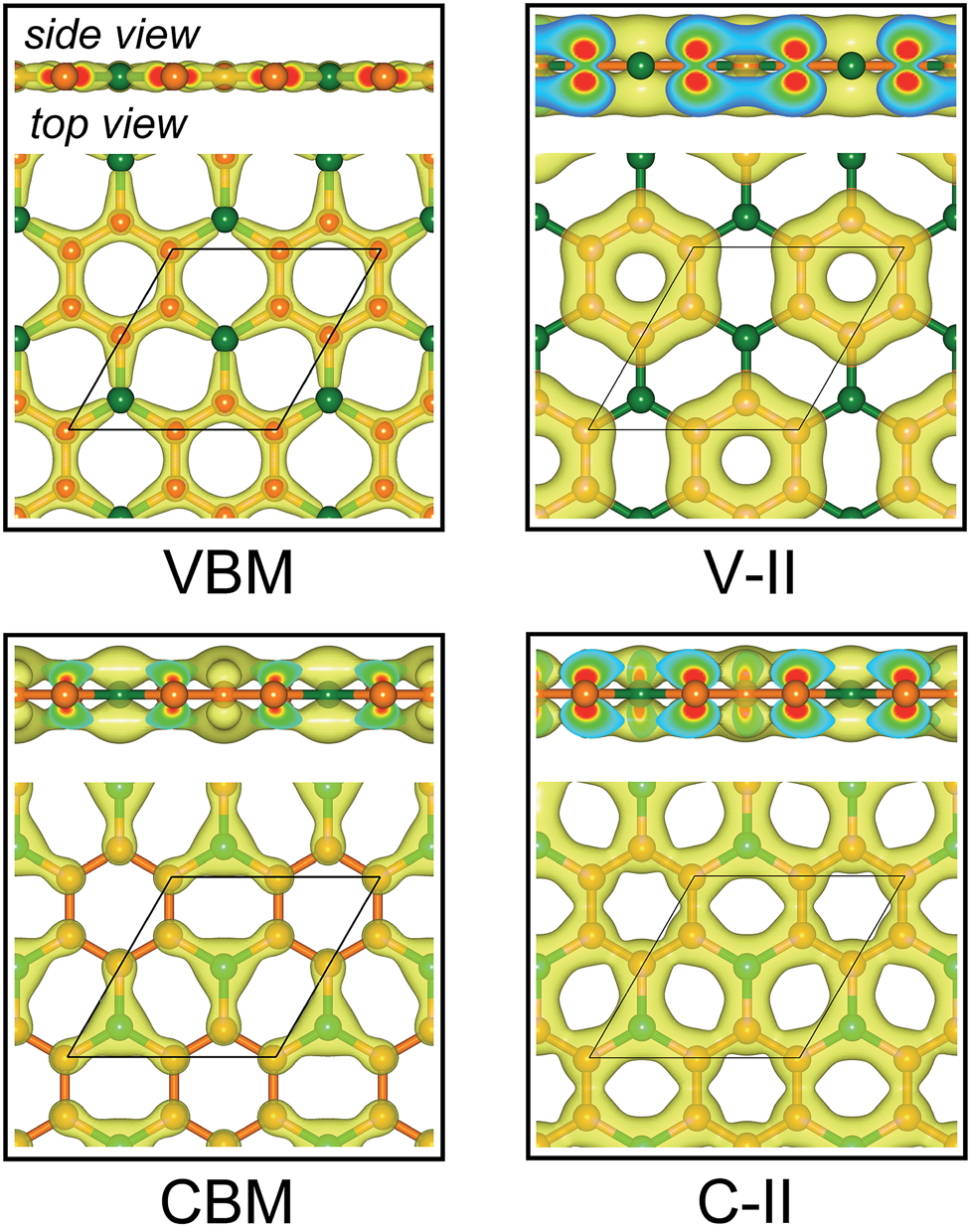
Spatial charge distribution of valence band maximum (VBM), conduction band minimum (CBM), V-II and C-II for BC_3_ monolayer. The points of V-II and C-II are marked out in Fig. 3a. Isosurface is set to 0.05 e Å^−3^. Orange and green balls represent C and B atoms.

Fig. 3a displays the electronic band structures of BC_3_ monolayer calculated by the HSE06 method. It can be seen that the BC_3_ monolayer is an indirect band gap semiconductor with the gap energy of 1.839 eV between the *Γ* and *M* points. In addition, using the PBE functional, an indirect band gap of 0.658 eV is also observed, which is much smaller than that predicted by the HSE06 method, but good agreement with the previous theoretical results.^20–23^ In views of the partial density of state (PDOS) and charge distribution shown in Fig. 3b and 4, the valence band maximum (VBM) is occupied by p_*x*_ and p_*y*_ orbitals of both B and C atoms, forming σ bonds of C–C and B–C in the whole layer. For the conduction band minimum (CBM), the π* anti-bonds formed by the p_*z*_ orbital between B and C atoms are the major characteristics. The corresponding charge is mainly distributed to BC_3_ molecules centered as the B atoms. As shown in Fig. 4, the level of π bonds from 75% graphene can still be observed at the V-II point, which is very close to the σ bonds at the VBM because of local distributions forming hexagonal rings. In deep energy levels, both types of the σ and π bonds can be formed in C–C and B–C bonds. Moreover, a Dirac point can be observed at the C-II point, which is contributed to the p_*z*_ orbitals of B and C atoms in the whole layer. As it is well known that graphene is zero gap semimetal. Without varying the hexagonal symmetry, an indirect band gap can be created in graphene by introducing B–C chemical interactions. Although the BC_3_ monolayer possesses a suitable band gap in the energy range of visible light (*i.e.*, corresponding to the wavelength of 675 nm or so), it may hold a poor efficiency in photoelectric conversion due to the spatial overlap of the charge distribution of VBM (hole) and CBM (electron).

**Fig. 5:**
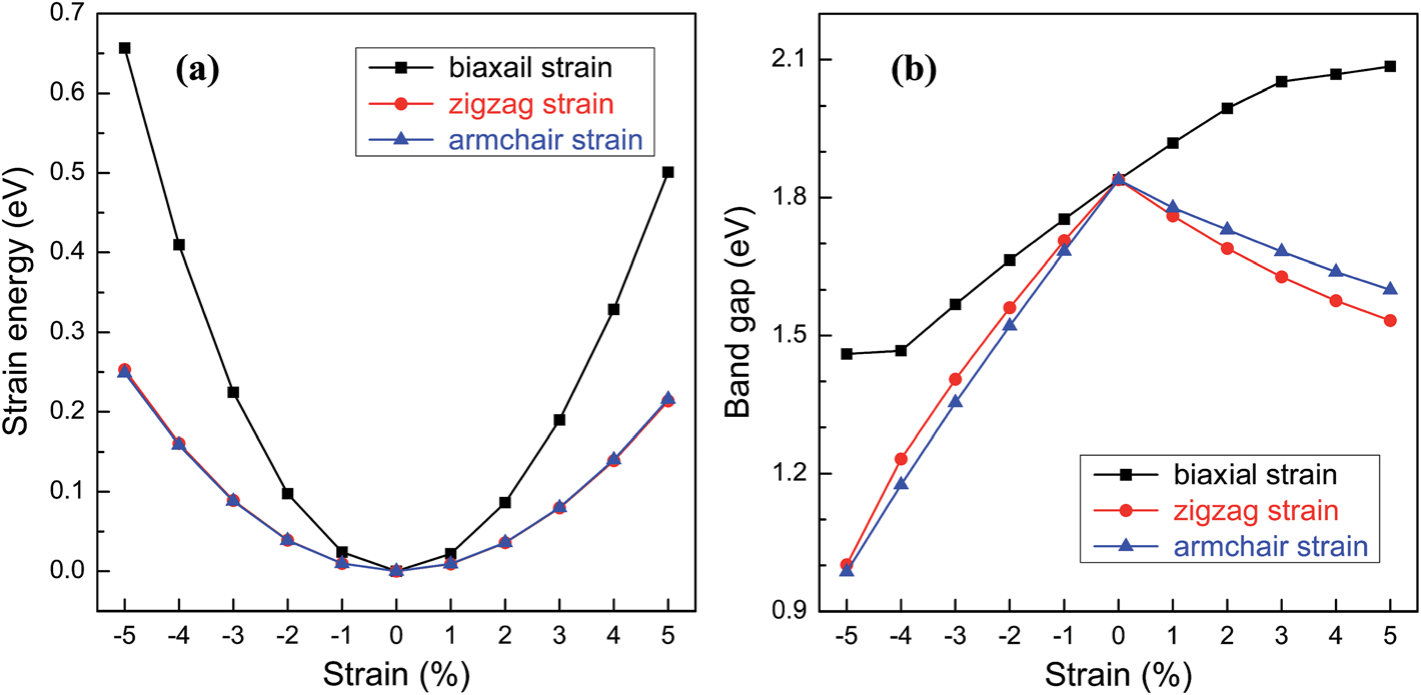
(a) Strain energy and (b) band gap of BC_3_ monolayer as a function of in-plane strains, *i.e.*, a biaxial strain and uniaxial strains applied along zigzag and armchair directions.

For practical applications in the electronic devices, tailoring the electronic properties is highly desirable. Even richer possibilities of the band structure engineering could be generated through applying in-plane strains to 2D nanostructures. Here, various in-plane strains, such as biaxial strain and uniaxial strains along zigzag and armchair directions are considered. Fig. 5a shows the relationship between the strain energy and the in-plane strain. Under the same strain, the strain energies along the zigzag and armchair directions are almost the same and much smaller than those of biaxial strain, suggesting that the structural deformation is more likely to occur along the uniaxial direction. The band gap of BC_3_ monolayer as a function of the strain is shown in Fig. 5b. Under the biaxial strain, a monotonic variation of the band gap is only observed. The band gap increases with the increasing tensile strain and decreases as the compressive strain increases with an average slope of 0.08 eV per strain. On the contrary, non-monotonic variations are presented when the uniaxial strain is applied to the BC_3_ monolayer. Despite of the zigzag and armchair strains, the band gap decreases monotonically as both the tensile and compressive strains increase. By comparison with the biaxial strain, a comparable slop is obtained under the uniaxial tensile strain, while a larger slop is presented by applying the uniaxial compressive strain. These results indicate that the BC_3_ monolayer exhibits sensitive tunability of band gap to the strain. As predicted by the previous reports,^45–47^ phosphorene is a direct band gap semiconductor with suitable gap energy of 1.6 eV or so. By applying various in-plane strains, its band gap can be tuned significantly. For example, a uniaxial tensile or compressive strain leads to a direct to indirect transition,^46^ which is independent of the strain direction. In this study, we have found that the BC_3_ monolayer is an indirect band gap semiconductor with moderate gap energy of 1.839 eV. Moreover, the relative large in-plane stiffness, highly dynamical stability, and flexible tunability of band gap enable the BC_3_ monolayer to hold great promise in nanoelectronic devices.

### Optical property

**Fig. 6:**
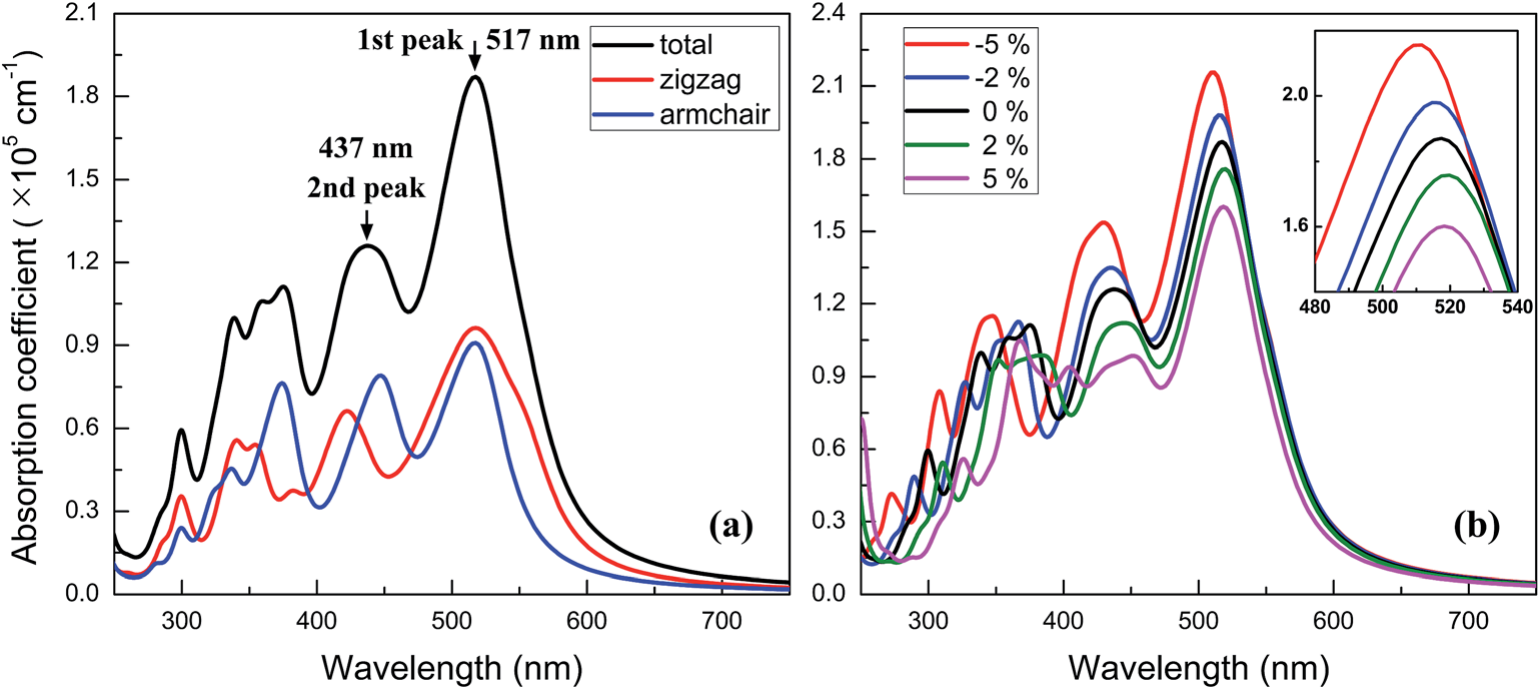
Spectra of optical absorption coefficient for (a) neutral and (b) biaxial strained BC_3_ monolayer.

Owning to the suitable band gap in the energy range of visible light spectrum, as well as the remarkable tunability of band gap, the BC_3_ monolayer could hold great promise for visible light harvesting. For exploring that, the properties of optical absorption are investigated by computing the complex dielectric function. The absorption coefficient *I*(*ω*) can be defined as following:^48^
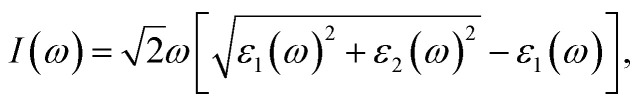
where *ε*_1_(*ω*) and *ε*_2_(*ω*) are the real and imaginary parts of the dielectric function, and *ω* is a given frequency. Moreover, the excitonic effects on optical properties are considered by using the time-dependent DFT (TDDFT) method.^49^ The spectra of optical absorption coefficient are shown in Fig. 6a. From this figure, it can be seen that the BC_3_ monolayer exhibits good visible light absorption. Two major characteristic peaks denoted as the 1st and 2nd ones are located at the wavelengths of 517 and 437 nm. Their absorption intensity is about a magnitude higher than that of phosphorene layer in the visible light range,^45^ and integrally declines as the wavelength decreases. Distinct from the phosphorene and its analogs,^45,50,51^ the anisotropy of optical absorption in the BC_3_ monolayer is very week, which can be inferred from the absorption spectra along the zigzag and armchair directions. Due to the large band gap (1.839 eV), little optical absorption is observed in the range of wavelength greater than 600 nm.

**Fig. 7:**
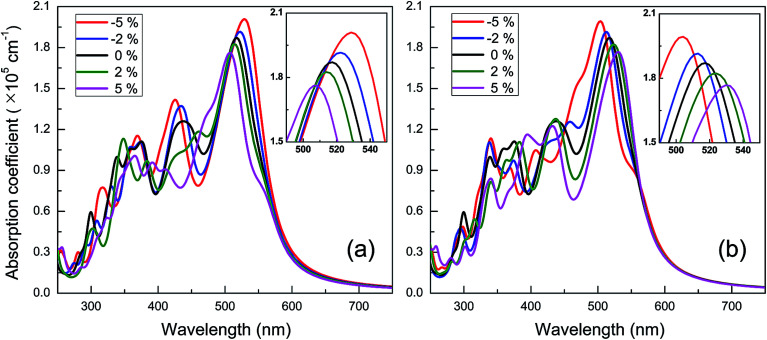
Spectra of optical absorption coefficient of BC_3_ monolayer under in-plane uniaxial strains along (a) zigzag and (b) armchair directions.

The spectra of optical absorption affected by various in-plane strains are explored. When an in-plane biaxial strain is applied to the BC_3_ monolayer, the major characteristic peaks of optical absorption vary significantly (see Fig. 6b). Their absorption intensity can be weakened by applying a tensile strain, while it can be enhanced by applying a compressive one. Independent of the biaxial strain, the 1st peak is located around the wavelength of 517 nm, exhibiting little red or blue shift. As the tensile and compressive strain increase, however, the 2nd peak exhibits obvious red and blue shifts, respectively. Besides, an in-plane uniaxial strain can also tune the optical absorption spectrum of the BC_3_ monolayer, as shown in Fig. 7. If the uniaxial strain is applied to different directions, various variations of the absorption spectra will be observed. Similar to the effects of biaxial strain, the intensity of both the 1st and 2nd peaks can be strengthened and weakened with the increasing compressive and tensile strains. In fact, the zigzag tensile and armchair compressive strains could result in the disappearance of the 2nd peak. Different from the biaxial strain, the zigzag compressive strain induces a red shift for the 1st peak, while the zigzag tensile strain leads to a blue shift (see Fig. 7a). Opposite trends of the 1st peak are presented when the armchair strain is applied (see Fig. 7b). Overall, the red or blue shift about 2.5 nm per strain is obtained in the 1st peak, which is independent of the zigzag and armchair strains. The results above suggest that the BC_3_ monolayer could exhibit flexible and strain-controllable optical properties, in terms of the intensity and wavelength of the absorption peak.

## Conclusions

In summary, we have performed a systematic research to explore the strain-tunable electronic and optical properties of BC_3_ monolayer, as well as the structural stability. The BC_3_ monolayer could be constructed from graphene by introducing B atoms to replace certain C atoms, exhibiting *P*6̄*mmm* space group. The results of phonon spectrum and *ab initio* MD simulation have verified that the BC_3_ monolayer is highly dynamic stable. The introduction of B atoms does not change the elastic property significantly, but can open an indirect band gap with the gap energy of 1.839 eV predicted by the HSE06 method. Such the indirect band gap is mainly attributed to the interactions of p–p orbitals among B and C atoms, forming σ bonds at the VBM and π* anti-bonds at the CBM. Furthermore, good visible light absorption is observed because of the moderate band gap corresponding to the energy of visible light spectrum. By applying various in-plane strains, the BC_3_ monolayer could exhibit the flexible tunability of band gap and optical absorption. Our results of good structural stability, moderate and tunable band gap, and strain-controllable optical absorption suggest that the BC_3_ monolayer holds great promise in the applications of nanoelectronic and optoelectronic devices.

## Conflicts of interest

There are no conflicts to declare.

## Supplementary Material
